# Incorporating kernelized multi-omics data improves the accuracy of genomic prediction

**DOI:** 10.1186/s40104-022-00756-6

**Published:** 2022-09-20

**Authors:** Mang Liang, Bingxing An, Tianpeng Chang, Tianyu Deng, Lili Du, Keanning Li, Sheng Cao, Yueying Du, Lingyang Xu, Lupei Zhang, Xue Gao, Junya Li, Huijiang Gao

**Affiliations:** Laboratory of Molecular Biology and Bovine Breeding, Institute of Animal Sciences, Chinese Academy of Agricultural Sciences, Beijing, 100193 People’s Republic of China

**Keywords:** BLUP, Cosine kernel, Genomic prediction, Transcriptome

## Abstract

**Background:**

Genomic selection (GS) has revolutionized animal and plant breeding after the first implementation via early selection before measuring phenotypes. Besides genome, transcriptome and metabolome information are increasingly considered new sources for GS. Difficulties in building the model with multi-omics data for GS and the limit of specimen availability have both delayed the progress of investigating multi-omics.

**Results:**

We utilized the Cosine kernel to map genomic and transcriptomic data as ${n}\times {n}$ symmetric matrix (***G*** matrix and ***T*** matrix), combined with the best linear unbiased prediction (BLUP) for GS. Here, we defined five kernel-based prediction models: genomic BLUP (GBLUP), transcriptome-BLUP (TBLUP), multi-omics BLUP (MBLUP, $\boldsymbol M=\mathrm{ratio}\times\boldsymbol G+(1-\mathrm{ratio})\times\boldsymbol T$), multi-omics single-step BLUP (mssBLUP), and weighted multi-omics single-step BLUP (wmssBLUP) to integrate transcribed individuals and genotyped resource population. The predictive accuracy evaluations in four traits of the Chinese Simmental beef cattle population showed that (1) MBLUP was far preferred to GBLUP (ratio = 1.0), (2) the prediction accuracy of wmssBLUP and mssBLUP had 4.18% and 3.37% average improvement over GBLUP, (3) We also found the accuracy of wmssBLUP increased with the growing proportion of transcribed cattle in the whole resource population.

**Conclusions:**

We concluded that the inclusion of transcriptome data in GS had the potential to improve accuracy. Moreover, wmssBLUP is accepted to be a promising alternative for the present situation in which plenty of individuals are genotyped when fewer are transcribed.

**Supplementary Information:**

The online version contains supplementary material available at 10.1186/s40104-022-00756-6.

## Background

A significant objective of genetics is to examine the connection between genotypes and phenotypes. Although genome-wide association studies (GWASs) have mapped thousands of common genetic variants for complex traits the causal variants and genes at these loci remain unknown [1]. This is because the mapping resolution is limited by the complicated linkage disequilibrium (LD) structure of the genome (i.e., the top associated variant at a locus is often not the causal variant) [2]. Especially for polygenic traits regulated by many interacting genes with minor effects [3], these detected significant loci could only explain a small proportion of phenotype variances, resulting in lower prediction accuracy.

Alternatively, genomic prediction (GP) is an ensemble of methods to estimate the breeding values with higher reliability earlier in life by combining DNA variants jointly using existing identification, pedigree, and phenotype databases for individuals [4, 5]. Over the past decades, this technology has revolutionized animal and plant breeding after its first implementation because of its excellent performance in reducing generation intervals and generating more genetic gain [6]. Currently, several methods have been made to develop more efficient statistical approaches to estimating genomic breeding values (GEBVs), such as genomic best linear unbiased prediction (GBLUP) which has been the most widely used in GP [7], single-step BLUP (ssGBLUP) [8], ridge regression methods [9], Bayesian Alphabet regression [10, 11], and emerging machine learning (ML) strategies including support vector regression (SVR) [12], random forest (RF) [13], reproducing kernel Hilbert spaces regression (RKHS) [10], kernel ridge regression [14], etc. Briefly, the predictive accuracy of Bayesian methods outperforms BLUP-based models in the majority of cases, while the Markov Chain Monte Carlo (MCMC) procedure also suffered a substantial computational burden [15]. In several simulation and actual studies, nonparametric ML methods behaved better, primarily due to their superior prediction ability [16, 17]. Therefore, there was a clear trend that increasingly breeders were trying to combine the advantages of multiple methods to estimate GEBVs in GP.

Afterward, one critical issue for phenotypic prediction is how to model non-additive effects (dominance or epistatic effects). Several research confirmed that using non-additive relationships that could improve the prediction of phenotypes [18, 19]. Incorporating additional layers of omics data into the prediction machine may partially solve this problem. For instance, many genetic variants affect complex traits by modulating gene expression, thus altering the abundance of relevant proteins [20]. The advanced next-generation sequencing technologies made it possible, and Li et al. discussed the concept of “omics-augmented broad sense heritability” that accounts for SNP-based effects and effects of downstream biological regulation captured by gene interactions [21]. However, the genetic links between phenotype and genome variants are too complex to determine directly at the genome level. Another concern was that the gene expression levels were affected by several factors such as tissue specificity, time of sampling and experimental conditions. Guo et al. found that using only transcriptome data to predict phenotypes is not satisfying, especially for high heritability traits; the genomic data remain the most efficient predictors [22]. Therefore, integrating multi-omics information could be a promising option in GP. In a human acute myeloid leukemia (AML) dataset including cytogenetics, gene mutations and expression variables, a priority-Lasso was presented and showed better predictive accuracy than the independent validation dataset [23]. Xu et al. found that the predictability of hybrid yield of rice can be further increased using multi-omics data, in particular, when used metabolomics data, the predictability was almost doubled compared with the genomic prediction [24].

**Fig. 1:**
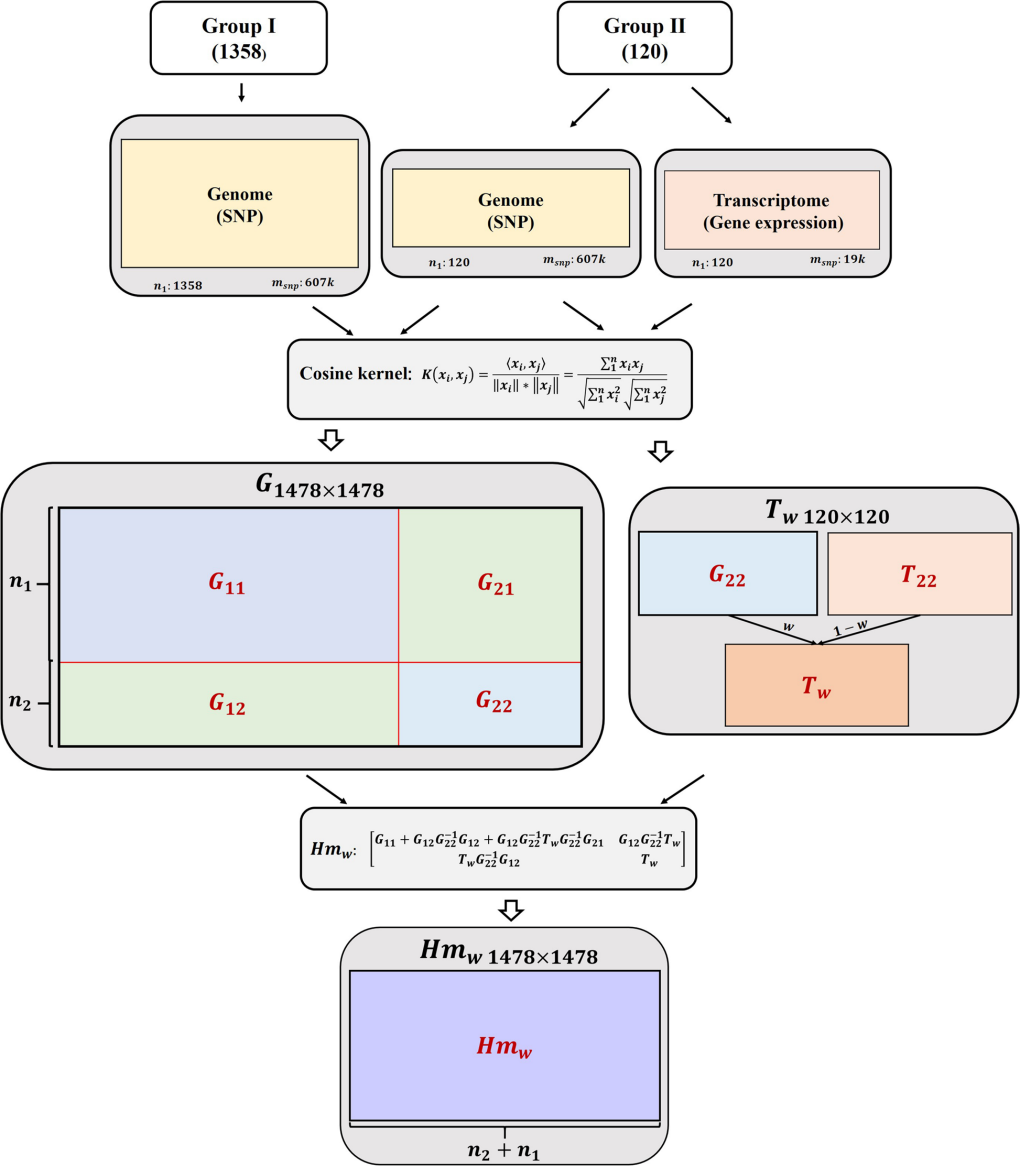
Flow charts of the three Cosine kernel-based methods. **a** In the experiment population, we defined MBLUP, where the $\boldsymbol M=\mathrm{ratio}\times\boldsymbol G+(1-\mathrm{ratio})\times\boldsymbol T$), and the ratio was weight parameter. **b** In the resource population, inspired by ssBLUP, we defined mssBLUP and wmssBLUP for solving the situation of fewer transcriptome data and more genome data

Based on our previous studies using the Cosine kernel transformed the SNP matrix of the population into an ${n}\times {n}$ (n is the size of the population) symmetric matrix, which is similar to the ***G*** matrix. So, we tried the Cosine kernel to map genomic and transcriptomic data as ${n}\times {n}$ symmetric matrix (***G*** matrix and ***T*** matrix). In this study, we firstly defined three prediction models: GBLUP (genomic data), TBLUP (transcriptomic data), and MBLUP (combining genomic and transcriptomic data, where the $\boldsymbol M=\mathrm{ratio}\times\boldsymbol G+(1-\mathrm{ratio})\times\boldsymbol T$) in experiment population (120 cattle both genotyped and transcribed). Additionally, large-scale studies systematically measuring the relationship between gene expression and a trait in individuals have been hampered because of the specimen availability and cost. We secondly construction of multi-omics single-step BLUP (mssBLUP) and weighted multi-omics single-step BLUP (wmssBLUP) to integrate transcribed individuals and genotyped resource population (Fig. 1) [5] inspired by $\boldsymbol H_{\boldsymbol w}$ matrix construction of single-step BLUP (ssBLUP) strategy. Genomic best linear unbiased prediction (GBLUP) using SNP array data was set to be a benchmark model (assessed only in resource population, 1478 cattle). Essentially, our research proposed an alternative strategy for integrating multi-omics data for genomic prediction, that is, to build a linear regression utilizing kernel trick mapped the original high-dimension data as a relationship matrix.

## Methods

All animals used in the study were treated following the Council of China Animal Welfare guidelines. Protocols of the experiments were approved by the Science Research Department of the Institute of Animal Sciences, Chinese Academy of Agricultural Sciences (CAAS), Beijing, China (approval number: RNL09/07).

### Data collection

#### Resource population

The Huaxi cattle population with an average age of 26 months and an average pre-slaughter weight of 700 kg were from Ulgai, Xilingol League, and Inner Mongolia of China. After weaning, all calves were moved to a fattening farm under uniform management and standardized feeding based on a total mixed ration (TMR) according to the eighth revised edition of nutrition requirement of beef cattle (NRC, 2016) [25]. Animals were slaughtered with electrical stunning, followed by bloodletting. The carcasses were chilled at 4 ℃ for 24 h, and about 1 kg longissimus dorsi muscle (LDM, 12-13th ribs) of the left side of the cold carcasses were sampled. After vacuum packing, all samples were stored at − 20 ℃ and transported to the laboratory for traits measurement.

#### Measurements of traits

We collected four traits for GP: longissimus dorsi muscles (LDM, kg), water holding capacity (WHC), shear force (SF, kg/N), and meat pH. The WHC was determined using TA-XT plus Texture Analyser 12,785 (Stable Micro Systems Ltd, Godalming, Surrey GU7 1YL, UK) according to reference NY/T 1333–2007 [26]. The SF was calculated following NY/T 1180–2006 method using a universal Warner–Bratzler testing machine MTS Synergie 200 (G-R Manufacturing Company, Trussville, AL, USA) [27], and the finally SF of each sample was the mean of three times testing. The pH of LDM was measured at about 24 h after slaughter by the pH meter HI 99,163 (HANNA Instruments, Woonsocket, RI, USA). The descriptive statistics of the phenotype are shown in Table 1.

**Table 1:** Descriptive statistics of phenotypes and heritability estimates for the four traits

Trait	*n*^a^	Mean ± SD	Maximum	Minimum	*h*^2^ ± SE
LDM, kg	1478	36.60 ± 8.79	68.12	17.06	0.18 ± 0.07
WHC, kg	1448	0.27 ± 0.04	0.38	0.07	0.13 ± 0.07
SF, kg	1457	5.58 ± 1.98	13.14	1.33	0.15 ± 0.05
pH	1478	5.55 ± 0.40	7.16	4.00	0.06 ± 0.06

*h*^*2*^ heritability, *SD* Standard deviation, *SE* Standard error

^a^Number of individuals with phenotype; *LDM* Longissimus dorsi muscle, *WHC* Water holding capacity, *SF* Shear force

#### Genotype and quality control

Genomic DNA was isolated from blood samples (1478 individuals) using the TIANamp Blood DNA Kit (Tiangen Biotech Co.Ltd., Beijing, Beijing, China). DNA quality was acceptable when the A260/A280 ratio was 1.8–2.0. All individuals were genotyped with an Illumina BovineHD BeadChip that contained 770,000 SNPs. Before quality control (QC), we removed the sex chromosome, the mitochondrial DNA, and unknown placement markers from the genotype. QC procedures were carried out using the PLINK v1.9 [28] to filter out SNPs with call rate < 90%, minor allele frequency (MAF) < 0.05, a significant deviation from the Hardy–Weinberg equilibrium (*P* < 10^–6^). Besides, the individuals with genotype missing rate greater than 10% were removed from the analysis. After testing QC criteria, 1478 cattle with 607,198 SNPs on 29 autosomal chromosomes with an average distance of 3 kb were included in subsequent studies. Among the resource population, all individuals were slaughtered between 2010 and 2021 when they were 18 to 24 months old, and 122 individuals were sampled for RNA extraction and transcriptome sequencing.

#### Total RNA extraction, library construction, and sequencing

LDM (12-13th ribs) was collected within 30 min after slaughter, and samples were immediately washed with phosphate-buffered saline (PBS) to avoid contamination. While slices of muscle tissues were saved into Eppendorf (EP) tubes and then frozen in liquid nitrogen. The total RNA was extracted by TRIzol reagent (Invitrogen, Life Technologies). Criteria of RNA concentration tested by Qubit® RNA Assay Kit (Life Technologies, Carlsbad, CA, USA), RNA purity tested by NanoPhotometer® spectrophotometer (Thermo Fisher Scientific, Waltham, MA, USA), and RNA integrity tested by RNA Nano 6000 Assay Kit of the Bioanalyzer 2100 system (Agilent Technologies, Santa Clara, CA, USA) were used to describe the total RNA quality. Presently, these samples (28S/18S > 1.8 and OD260/280 ratio > 1.9) were used to construct cDNA libraries and performed RNA sequencing when the RNA integrity number (RIN) > 7. The cDNA library construction was generated by IlluminaTruSeqTM RNA Kit (Illumina, San Diego, CA, USA), and the RNA sequencing was performed on an Illumina NovaSeq 6000 platform by paired-end strategy (read length 150 bp).

#### QC for RNA sequencing data

To obtain clean reads, MD5 values reflected the integrity of the raw sequencing reads, and FastQC (v0.11.9) assessed the quality of the reads in terms of base composition and quality distribution [29]. All sequencing results were visualized by MultiQC (v1.9) [30]. Reads containing ploy-N (the percentage of undetermined base information > 5% in a read), trimmed adapters, and low-quality reads were discarded using Trimmomatic (v0.39) [31].

#### Reads mapping

HISAT2 (v2.2.1) was used to compare clean reads to reference the genome *Bos taurus* ARS-UCD1.2 [32]. The cattle reference genome annotation supplied the genomic position information was used to calculate effective reads aligned to the gene region. File generated by the HISAT2 were sorted through SAMtools (v1.11) [33, 34]. FeatureCounts (v1.5.2) was used to estimate read counts generated from RNA sequencing experiments [35]. After the above process, we obtained the original reads matrix (120 cattle, 27,607 genes), 7546 genes with 0 reads in all individuals and 1007 genes located on sex chromosomes were removed. Then, using STRINGTIE software (v.1.3.4 with default settings), the expression levels of genes (fragments per kilobase of transcript per million mapped reads, FPKM) were calculated. Genes with FPKM < 0.1 in more than 95% of samples were discarded (*n* = 3729) [36]. After this quality control step, a total of 15,325 gene expression transcripts from 120 cattle remained for further analysis.

### Genomic prediction comparison models

To remove the fixed effects in prediction, we used adjusted phenotypic values of phenotypes in subsequent analysis:

$$\boldsymbol y=\boldsymbol X\boldsymbol \beta +{y}^{*}$$

where ***y*** is a vector of phenotypic, $\boldsymbol \beta$ is a vector of fixed effects (year of birth, birth weight, fattening duration, and slaughtered batch as a covariate), ***X*** is the design matrix of relevant observations, and ${y}^{*}$ is the random residual, which was subsequently used in the prediction models.

#### Genomic best linear unbiased prediction (GBLUP)

GBLUP assumes that all SNPs contribute to the genetic variance and follow the same normal distribution [7]. GEBVs were calculated based on the following equation:

$$\boldsymbol y^\ast=\boldsymbol Z\gamma+\boldsymbol e\;with\;\boldsymbol \gamma\sim N\left(0,\boldsymbol G\sigma_g^2\right)\;and\;\boldsymbol e\sim N\left(0,\boldsymbol I\sigma_e^2\right)$$

where $\boldsymbol{y}^{*}$ is the vector of the corrected phenotype and ***Z*** is an incidence matrix for individual effects. $\boldsymbol\gamma$ is a vector of breeding values and ${\sigma }_{g}^{2}$ is genetic variance. $\boldsymbol e$ is a vector of residual error, where $\boldsymbol I$ is an identity matrix and ${\sigma }_{e}^{2}$ is the residual variance. Hence, the narrow sense of heritability was estimated by the formula: ${h}^{2}=\frac{{\sigma }_{g}^{2}}{{\sigma }_{g}^{2}+{\sigma }_{e}^{2}}$. The ***G*** matrix was calculated as $\boldsymbol G=\frac{Z{Z}^{^{\prime}}}{2\sum {p}_{i}(1-{p}_{i})}$, and ${p}_{i}$ is the MAF of the *i*-th marker [7].

#### Kernel trick

Based on our previous study, the kernel matrix transformed by Cosine kernel was analogous to the numerator relationship ***G*** matrix, which had a well-matched performance for the ***G*** matrix, with time consumption reduced by 20 times [37]. In this research, we choose the Cosine kernel to transform original genomic and transcriptomic data:

$$K\left({x}_{i},{x}_{j}\right)=\frac{\langle {x}_{i},{x}_{j}\rangle }{\Vert {x}_{i}\Vert *\Vert {x}_{j}\Vert }=\frac{{\sum }_{1}^{n}{x}_{i}{x}_{j}}{\sqrt{\sum_{1}^{n}{x}_{i}^{2}}\sqrt{\sum_{1}^{n}{x}_{j}^{2}}}$$

For the ***G*** matrix, ${x}_{i}$ and ${x}_{j}$ were the feature vectors of individual *i* and *j* in an m-dimensional feature space, respectively, where m is the number of SNPs. For the T matrix, ${x}_{i}$ and ${x}_{j}$ were the $1\times\boldsymbol n$ vector and was kernelized in an n-dimensional feature space, where n is the number of genes. The ***G*** matrix and ***T*** matrix were measured by the cosine of the angle between two vectors, and the regularization factor C was determined by gird search as 0.05 in our previous study.

#### Multi-omics best linear unbiased prediction (MBLUP)

For experimental populations who have both genomic and transcriptomic data (120 individuals), we defined an MBLUP, where the ***M*** matrix was used to replace the ***G*** matrix in traditional GBLUP equations. Here, the $\boldsymbol M=\mathrm{ratio}\times\boldsymbol G+(1-\mathrm{ratio})\times\boldsymbol T$) and the gradient of weight parameter ratio were set as 0.01–0.99 to gain the optimum predictive accuracies for each trait. The MBLUP was equivalent to TBLUP when the ratio was 0 and was comparable to GBLUP when the ratio was 1.

#### Multi-omics single-step best linear unbiased prediction (mssBLUP)

Inspired by the single-step best linear unbiased prediction (ssBLUP) [5]:

$$\boldsymbol{H}=\left[\begin{array}{cc}\boldsymbol{H}_{\boldsymbol{11}}& \boldsymbol{H}_{\boldsymbol{12}}\\ \boldsymbol{H}_{\boldsymbol{21}}& \boldsymbol{H}_{\boldsymbol{22}}\end{array}\right]=\left[\begin{array}{cc}\boldsymbol{A}_{\boldsymbol{11}}+\boldsymbol{A}_{\boldsymbol{12}}\boldsymbol{A}_{\boldsymbol{22}}^{\boldsymbol{-1}}\boldsymbol{A}_{\boldsymbol{21}}+\boldsymbol{A}_{\boldsymbol{12}}\boldsymbol{A}_{\boldsymbol{22}}^{\boldsymbol{-1}}\boldsymbol{G}\boldsymbol{A}_{\boldsymbol{22}}^{\boldsymbol{-1}}\boldsymbol{A}_{\boldsymbol{21}}& \boldsymbol{A}_{\boldsymbol{12}}\boldsymbol{A}_{\boldsymbol{22}}^{\boldsymbol{-1}}\boldsymbol{G}\\ \boldsymbol{G}\boldsymbol{A}_{\boldsymbol{22}}^{\boldsymbol{-1}}\boldsymbol{A}_{\boldsymbol{21}}& \boldsymbol{G}\end{array}\right]$$

the inverse of $\boldsymbol{H}^{-1}$:

$$\boldsymbol{H}^{\boldsymbol{-1}}=\boldsymbol{A}^{\boldsymbol{-1}}+\left[\begin{array}{cc}0& 0\\ 0& \boldsymbol{G}^{-1}-\boldsymbol{A}_{\boldsymbol{22}}^{\boldsymbol{-1}}\end{array}\right]$$

To address the issue that large numbers of individuals were genotyped but fewer are transcribed, here, we built multi-omics single-step best linear unbiased prediction (mssBLUP):

$$\boldsymbol{Hm}=\left[\begin{array}{cc}\boldsymbol{Hm}_{\boldsymbol{11}}& \boldsymbol{Hm}_{\boldsymbol{12}}\\ \boldsymbol{Hm}_{\boldsymbol{21}}& \boldsymbol{Hm}_{\boldsymbol{22}}\end{array}\right]=\left[\begin{array}{cc}\boldsymbol{G}_{\boldsymbol{11}}+\boldsymbol{G}_{\boldsymbol{12}}\boldsymbol{G}_{\boldsymbol{22}}^{\boldsymbol{-1}}\boldsymbol{G}_{\boldsymbol{21}}+\boldsymbol{G}_{\boldsymbol{12}}\boldsymbol{G}_{\boldsymbol{22}}^{\boldsymbol{-1}}\boldsymbol{T}\boldsymbol{G}_{\boldsymbol{22}}^{\boldsymbol{-1}}\boldsymbol{G}_{\boldsymbol{21}}& \boldsymbol{G}_{\boldsymbol{12}}\boldsymbol{G}_{\boldsymbol{22}}^{\boldsymbol{-1}}\boldsymbol{T}\\ \boldsymbol{T}\boldsymbol{G}_{\boldsymbol{22}}^{\boldsymbol{-1}}\boldsymbol{G}_{\boldsymbol{21}}& \boldsymbol{T}\end{array}\right]$$

where $T$ was as mentioned above. The ${\boldsymbol G}_{11}$ was the submatrix of ***G*** for transcribed animals. The ${\boldsymbol G}_{22}$ was the submatrix of ***G*** for non-transcribed animals. The ${\boldsymbol G}_{12}$ (or ${\boldsymbol G}_{21}$) was the submatrix of ***G*** describing the relationships between transcribed and non-transcribed animals.

#### Weighted multi-omics single-step best linear unbiased prediction (wmssBLUP)

For higher accuracy, several research studies weighted the ***G*** matrix in ssBLUP, in which the ${\boldsymbol G}_w=\left(1-w\right)\boldsymbol G+w{\boldsymbol A}_{22}$, the $w$ is a weighted parameter, which indicated the proportion of genetic relationships that were not explained by SNPs [38–40]. Afterward, we built the weighted multi-omics single-step linear unbiased prediction (wmssBLUP):

$$\boldsymbol{Hm}_{\boldsymbol{w}}=\left[\begin{array}{cc}\boldsymbol{Hm}_{\boldsymbol{11}}& \boldsymbol{Hm}_{\boldsymbol{12}}\\ \boldsymbol{Hm}_{\boldsymbol{21}}& \boldsymbol{Hm}_{\boldsymbol{22}}\end{array}\right]=\left[\begin{array}{cc}\boldsymbol{G}_{\boldsymbol{11}}+\boldsymbol{G}_{\boldsymbol{12}}\boldsymbol{G}_{\boldsymbol{22}}^{\boldsymbol{-1}}\boldsymbol{G}_{\boldsymbol{21}}+\boldsymbol{G}_{\boldsymbol{12}}\boldsymbol{G}_{\boldsymbol{22}}^{\boldsymbol{-1}}\boldsymbol{T}_{\boldsymbol{w}}\boldsymbol{G}_{\boldsymbol{22}}^{\boldsymbol{-1}}\boldsymbol{G}_{\boldsymbol{21}}& \boldsymbol{G}_{\boldsymbol{12}}\boldsymbol{G}_{\boldsymbol{22}}^{\boldsymbol{-1}}\boldsymbol{T}_{\boldsymbol{w}}\\ \boldsymbol{T}_{\boldsymbol{w}}\boldsymbol{G}_{\boldsymbol{22}}^{\boldsymbol{-1}}\boldsymbol{G}_{\boldsymbol{21}}& \boldsymbol{T}_{\boldsymbol{w}}\end{array}\right]$$

where the ${\boldsymbol T}_w=\left(1-w\right)\boldsymbol T+w{\boldsymbol G}_{22}$, the $w$ is a weighted parameter between 0 and 1, which indicated the proportion of genetic relationships that were not explained by gene expression levels.

The inverse of $\boldsymbol{{Hm}_{w}}$:

$$\boldsymbol{{Hm}_{w}^{-1}}=\boldsymbol{{G}^{-1}}+\left[\begin{array}{cc}0& 0\\ 0& \boldsymbol{{T}_{w}^{-1}}-\boldsymbol{{G}_{22}^{-1}}\end{array}\right]$$

Here, the gradient of parameter $w$ was set as 0–1 to gain the optimum predictive accuracies for Chinese Simmental beef cattle.

### Assessing prediction performance

For experimental populations with transcriptome data (120 individuals), because of the limited group size, we adopt Leave One Out (LOO) to assess the predictive performance of GBLUP, MBLUP, and TBLUP. LOO is simple cross-validation (CV), which generated lower generalization errors than cross-validation in the small-scale population [41]. Each learning set is created by taking all the samples except one, the test set being the sample left out. Thus, for 120 samples, 119 individuals are used as the training set to train the model, and the remaining one is used as the test which was predicted using the trained model. Repeat the above process 120 to ensure that each individual is used as the training set once. And then, predictive accuracies were expressed Pearson’s correlation between 120 GEBVs and ${y}^{*}$. The formula was as follows: $r({y}^{*}, GEBV)=\frac{cov({y}^{*}, GEBV)}{\sqrt{var({y}^{*})}var\sqrt{GEBV}}$, where ${y}^{*}$ was the corrected phenotype.

For the resource population (1478 individuals), the predictive accuracies of GBLUP (based SNP data, assessed only in resource population), mssBLUP, and wmssBLUP were quantified with Pearson’s correlation based on five replicates of fivefold CV. The predictive accuracy performance of each method was the average Pearson correlation of 5 replicates in the validation subset.

## Results

### Compared performance between GBLUP, MBLUP, and TBLUP

**Fig. 2:**
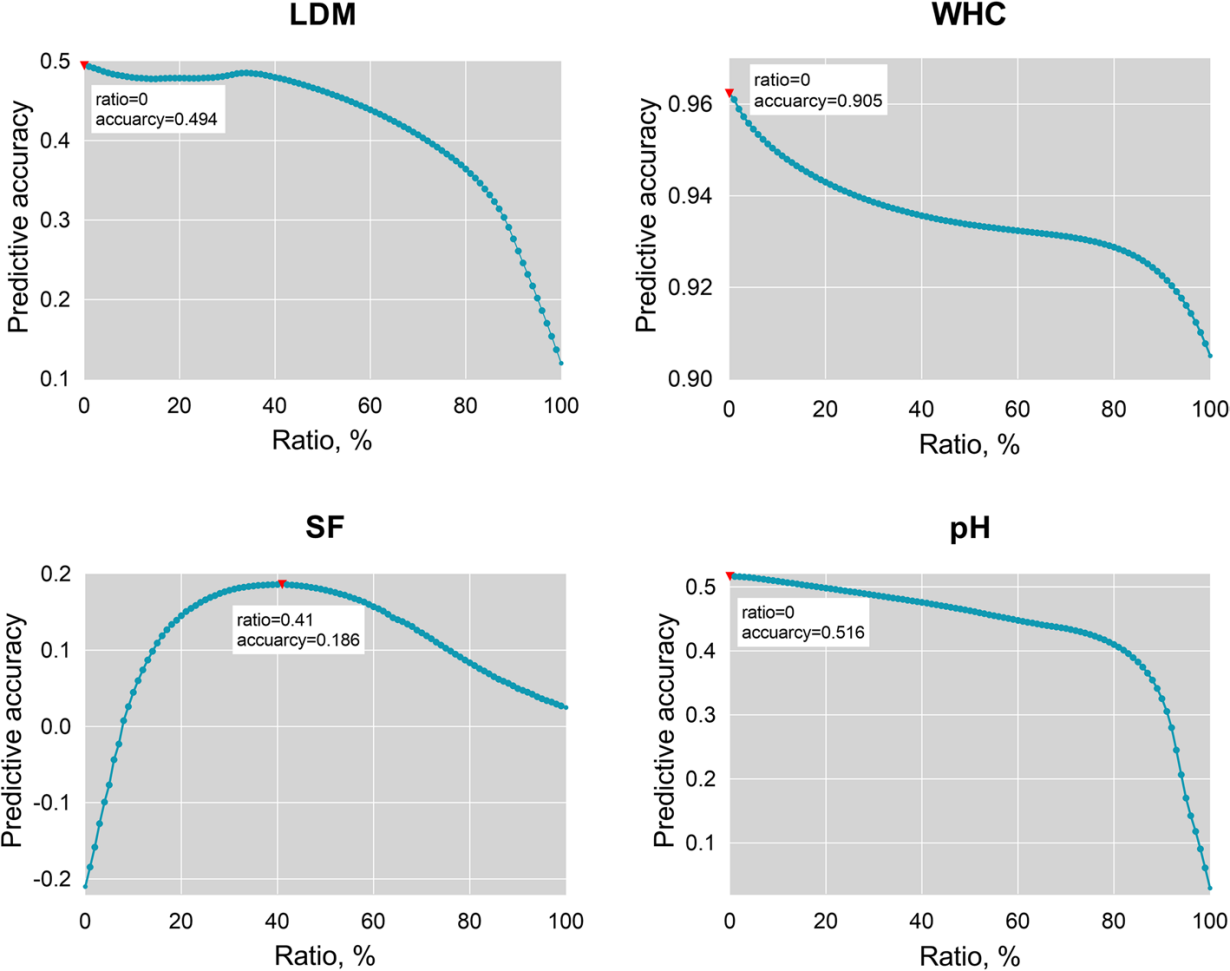
The predictive accuracies of MBLUP under different radios (where the ratio was set as 0.01–0.99, and the $\boldsymbol M=\mathrm{ratio}\times\boldsymbol G+(1-\mathrm{ratio})\times\boldsymbol T$) for four traits in the experiment population (120 cattle). The accuracies were assessed by LOO. for 120 samples, and we have 120 different training sets and 120 different test sets. And then, predictive accuracies were expressed Pearson’s correlation between 120 GEBVs and ${y}^{*}$

The weighting parameter ratio in MBLUP was set with a gradient of 0.01–0.99 for determining the optimal coefficient for the ***M*** matrix. The predictive accuracies of MBLUP for four traits in the experiment population are shown in Fig. 2. The predictive accuracy of LDM, HWC, and pH traits has the same changing trend, with gradually decreased along with the increase of ratio value. When the ratio was 0, the MBLUP (or TBLUP) was far preferred to GBLUP. The predictive trend of the SF trait first increased and then decreased, reaching the maximum when the ratio was 0.41.

### Predictive performance of GBLUP, mssBLUP, and wmssBLUP

**Fig. 3:**
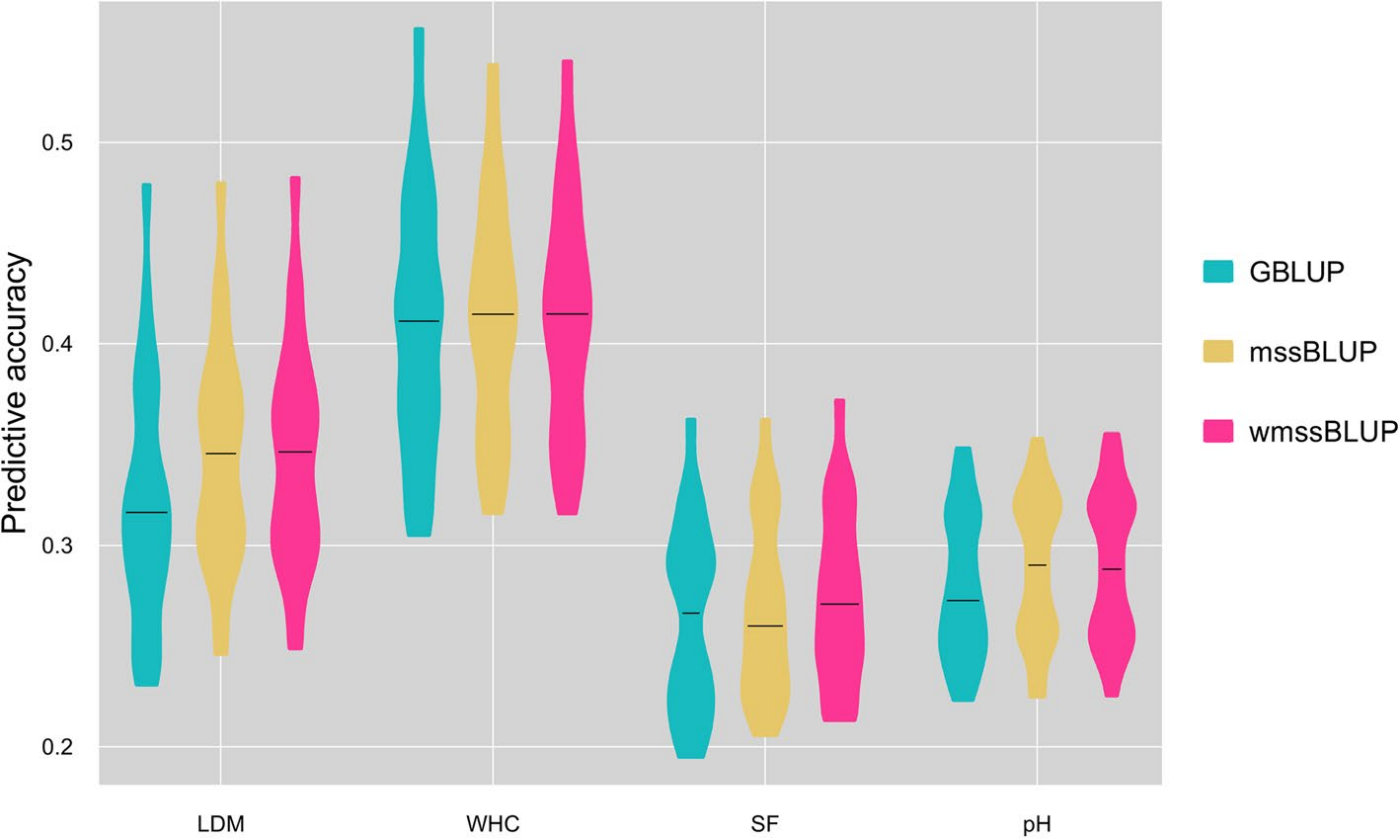
Comparison of prediction accuracy performances of GBLUP, mssBLUP, and wmssBLUP. For wmssBLUP, the $w$ was 0.1, 0.6, 0.5, and 0.4, respectively. The prediction accuracy performance of each method was measured by the average Pearson correlation between predicted values and phenotypic values of 5 replicate in the validation subset. In each replicate, the dataset was randomly split into a reference subset containing 80% of individuals and a validation subset containing the remaining 20%. For each violin plot, the middle line represents the median value, and the upper and lower ends of each box represent the maximum and minimum

For wmssBLUP, the gradient of weight parameter *w* was set as 0–1 to explore the optimum predictive accuracies for each trait. When $w$ was 0.1, 0.6, 0.5, and 0.4, respectively, the predictive ability of wmssBLUP reached its peak (Fig. S1). So, we used this group weighted value to assess the predictive performance of GBLUP, mssBLUP, and wmssBLUP for four traits (Fig. 3). The details of 25 Pearson’s correlation coefficients of GBLUP, mssBLUP, and wmssBLUP were listed in Table S1. The mssBLUP outperformed GBLUP for all four evaluated traits, and the average improvement was 3.37%, of which the accuracy improvement of LDM traits was the most obvious, reaching 7.50%. The wmssBLUP performed best among three models for all traits, while the average improvement of which over GBLUP was 4.18%, and over mssBLUP was 0.79%.

### The proportion of transcribed population influenced the wmssBLUP

**Fig. 4:**
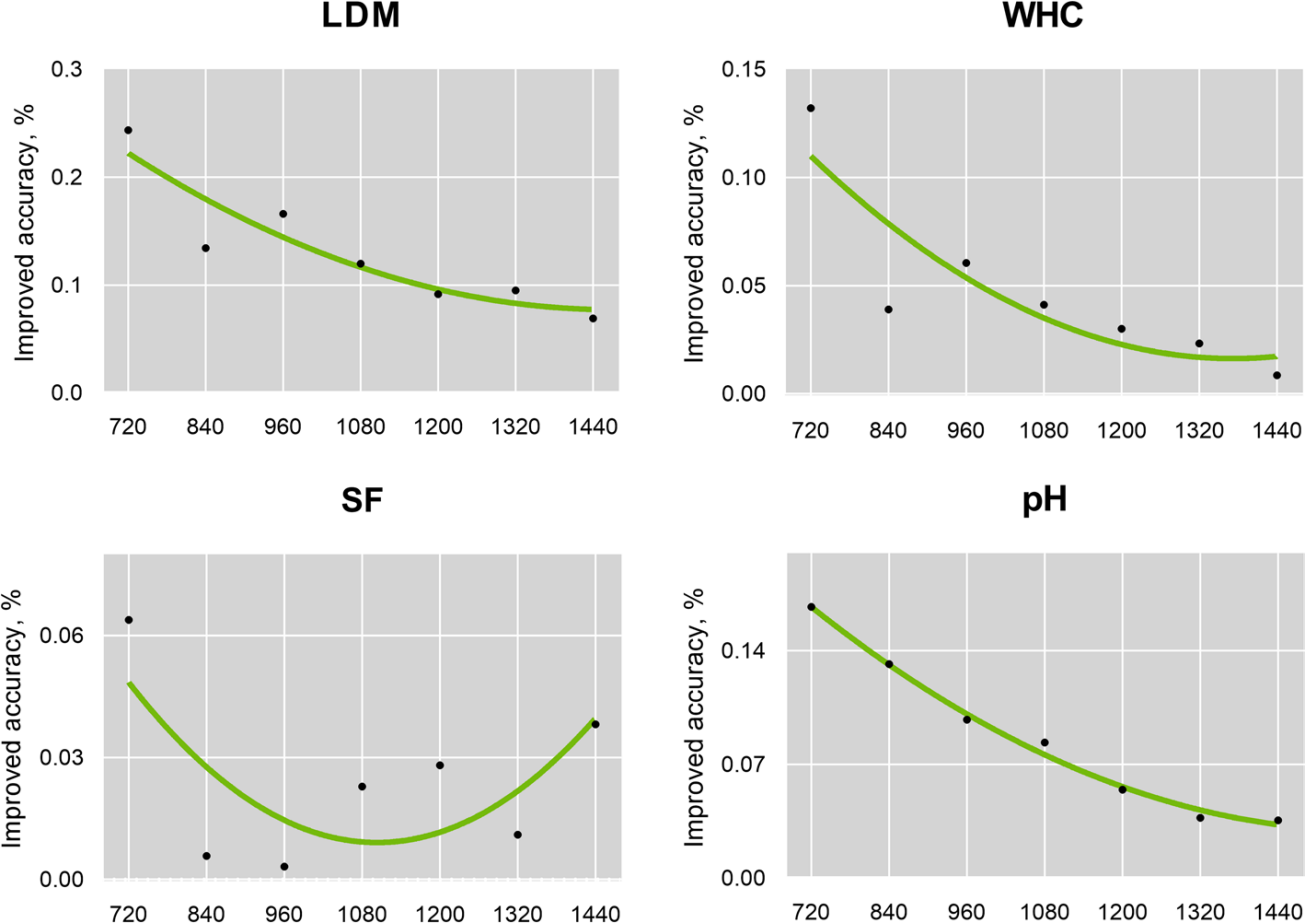
The average percentage improvement of wmssBLUP over GBLUP in different population scales. The measure of prediction accuracies was consistent with Fig. 3

Additionally, except for selecting the optimum weighted parameter *w*, we also considered the influence of the proportion of transcribed data in the whole resource population on the statistical power of the wmssBLUP. Based on the weighted value *w* for each trait mentioned above, firstly, 600, 720, 840, 960, 1080, 1200, and 1320 individuals from the resource population (1358 cattle with genotype only) were randomly extracted, respectively. And then combined the selected groups with the experimental population (120 cattle with both genotyped and transcribed) to build the ${\boldsymbol H\boldsymbol m}_{\boldsymbol w}$ matrix and evaluated the prediction accuracy of wmssBLUP, respectively. The details of accuracies were listed in Table S2 which demonstrated the trend of the accuracy level of wmssBLUP with the group size. The accuracy of wmssBLUP and GBLUP showed an improved trend with increasing population scale (Fig. S2). However, the wmssBLUP consistently outperformed GBLUP in all cases, and the average improvement was 13.14%, 4.78%, 2.47%, and 8.67% for four traits, respectively. As seen in Fig. 4, the improved accuracy of the wmssBLUP over the GBLUP was getting smaller with decreasing proportion of transcribed cattle in the whole resource population (from 15.16% to 3.04%).

## Discussion

Genomic selection (GS) is a promising method in modern molecular breeding practice because of its demonstrated effectiveness in predicting accuracies and shortening generation intervals. As a significant advance of next-generation sequencing, transcriptome and other omics data provide new information sources for phenotypic prediction. Meanwhile, the current progress of GS using genomic data alone has reached a bottleneck and integrating multi-omics as a novel predictive factor analysis may be a promising way to improve accuracies [42]. Based on our previous study, An et al. [37] defined a Cosine similarity matrix (CS matrix), which was generated in the process of kernelizing the term-frequency of the minor allele (0, 1, and 2) in high-dimensional feature space. The results showed the CS matrix had a well-matched performance for the ***G*** matrix (calculated by VanRaden [7]) with a computational efficiency increase by 20 times. In this research, we used the Cosine trick to kernelize the genome and transcriptome data to an ${n}\times {n}$ relationship matrix, named ***G*** and ***T*** matrix (distinct from the traditional ***G*** matrix of VanRaden). Afterward, the proposition of integrating multiple omics is transformed into the conventional calculation problem of matrixes. Therefore, we evaluated three kernel-based investigating methods with the above foundation: MBLUP, mssBLUP, and wmssBLUP.

For MBLUP in this study, we set a weighted parameter, valued between 0 and 1, aimed to explore an optimal ratio for each trait. The reported studies had proven that the resource population scale significantly determined the reliability of genomic predictions [43, 44]. Due to the reason that the population size of MBLUP was 120, the accuracy of HWC and SF appeared in some unusual values, actually the accuracy of SF was 0.28 in general [45]. Here, we mainly emphasized the effects of modeling transcriptome data into the prediction model, that was, comparing the trend of MBLUP, GBLUP (ratio = 1), and TBLUP (ratio = 0). This matched those observed in earlier studies [46, 47]. Typically, in this small experiment population with limited condition and cost, participation of gene expression data in GS had the potential to improve genomic predictions.

Before the genotyping technology matured a decade ago, GP was limited by the number of animals for which both genotypes and phenotypes were available. A single-step BLUP (ssBLUP) was proposed to predict GEBV using information from genotype and pedigree simultaneously [48]. The weighted single-step BLUP (wssBLUP) was derived from ssBLUP. This model was more accurate by using a weighted scaled and properly augmented relationship matrix ($\boldsymbol H$ matrix) [49, 50]. Similarly, integrating multi-omics for GP faced the dilemma that large-scale studies systematically measuring the association between omics data and traits have been hampered because of the specimen availability and cost. Therefore, we developed mssBLUP and wmssBLUP, in which the $\boldsymbol H\boldsymbol m$ and ${\boldsymbol H\boldsymbol m}_w$ matrix both were constructed by kernel-based ***G*** and ***T*** matrix. In this study, compared with traditional GBLUP, wmssBLUP and mssBLUP had 4.18% and 3.37% average improvement of predictive accuracy. And the weighted coefficient *w* changed with the data structure. This is also in agreement with our earlier observations, which showed that predictive ability can be improved when combining transcripts with SNPs, but it depends on the traits [51]. For another, the mssBLUP and wmssBLUP appeared to be more reliable and robust than MBLUP. While the degree of improvement of wmssBLUP decreased with the lessening proportion of transcribed cattle in the whole resource population (Fig. S2). These results corroborate the findings of a great deal of the previous work in GBLUP and ssBLUP, that was modeling increasing predictive factors, including pedigree information, genome, transcriptome, and metabolic data, even intergenic, gene, exon, protein-coding sequences et al. showed a common tendency that could partly contribute to the improvements of phenotype prediction [6, 52, 53]. Therefore, we conclude that mssBLUP and wmssBLUP will be promising alternatives for the current reality of genomic prediction with fewer omics data but far more SNP array.

Therefore, future studies on the current topic are recommended: 1) One such decision concerns which suitable kernel to use. Studies found that taking the Gaussian kernel mapping transcriptome data for predicting phenotypes gained little effect [21]. 2) The complex interaction of multi-omics should be modelled, while consider the overfitting problem. Xu et al. observed that the predictive ability decreased when combining transcriptome and metabolic data into GP for six yield-related traits in maize [54]. 3) In addition, a rising concern was that the gene expression levels were affected by several factors, such as tissue specificity, time of sampling, and experimental conditions. It is necessary to balance the costs and genetic gains of using transcriptomic in-formation in genomic predictions. Essentially, we emphasize the feasibility of this opinion, that is, the kernel algorithm is taken to map the original data into an ${n}\times {n}$ relationship matrix and then build linear regression with the phenotypes.

## Conclusions

In the present study, we proposed three Cosine kernel-based methods to investigate multi-omics data: MBLUP, mssBLUP, and wmssBLUP. Our results showed MBLUP was far preferred to GBLUP (ratio = 1) in four traits. While, wmssBLUP and mssBLUP outperformed GBLUP, and the average improvement was 4.18% and 3.37%. We also found the transcriptome data has the potential to improve genomic predictions if they can be included on a larger scale. Our research proposes reliable and robust alternatives for the present situation in that large numbers of individuals were genotyped, but fewer were transcribed.

## Supplementary Information

**Additional file 1:** **Fig. S1.** The determination of the weight parameter w in wmssBLUP.

**Additional file 2:** **Fig. S2.** The comparison of the accuracy of wmssBLUP and GBLUP in different population scales.

**Additional file 3:** **Table S1.** The details of 25 Pearson’s correlation coefficients of GBLUP, mssBLUP, and wmssBLUP for LDM, HWC, SF, and pH.

**Additional file 4:** **Table S2.** The summary of the accuracy of wmssBLUP and GBLUP for increasing population scale.

## Data Availability

The datasets used during the current study are available from the corresponding authors on reasonable request. The genotype datasets are available from the Dryad Digital Repository https://datadryad.org/stash/dataset/doi:10.5061/dryad.4qc06.

## Abbreviations

BLUP: Best linear unbiased prediction
CV: Cross-validation
GBLUP: Genomic BLUP
GEBVs: Genomic breeding values
GP: Genomic prediction
GS: Genomic selection
GWASs: Genome-wide association studies
LD: Linkage disequilibrium
LDM: Longissimus dorsi muscle
mssBLUP: Multi-omics single-step BLUP
MAF: Minor allele frequency
MBLUP: Multi-omics BLUP
MCMC: Markov Chain Monte Carlo
ML: Machine learning
QC: Quality control
RF: Random Forest
RKHS: Reproducing kernel Hilbert spaces regression
SVR: Support vector regression
ssGBLUP: Single-step BLUP
SF: Shear force
wmssBLUP: Weighted multi-omics single-step BLUP
WHC: Water holding capacity
