# Supplementary figures and images for: Incorporating kernelized multi-omics data improves the accuracy of genomic prediction
Source: J Anim Sci Biotechnol. 2022 Sep 20;13:103. doi: 10.1186/s40104-022-00756-6 (PMC9490992; doi:10.1186/s40104-022-00756-6)

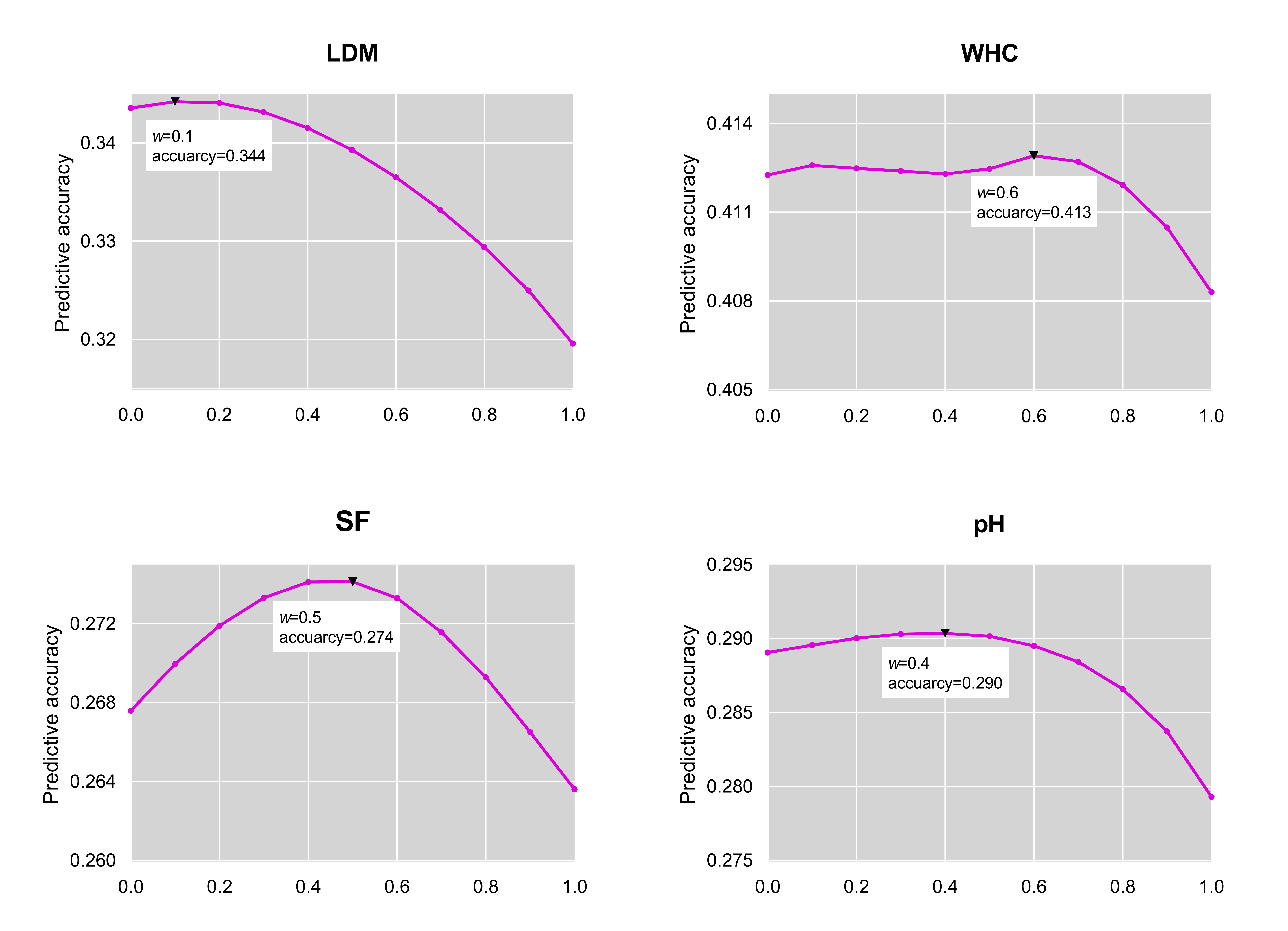

Supplement: Supplementary file 1 — Additional file 1: Fig. S1. The determination of the weight parameter w in wmssBLUP. [file 40104_2022_756_MOESM1_ESM.tif]

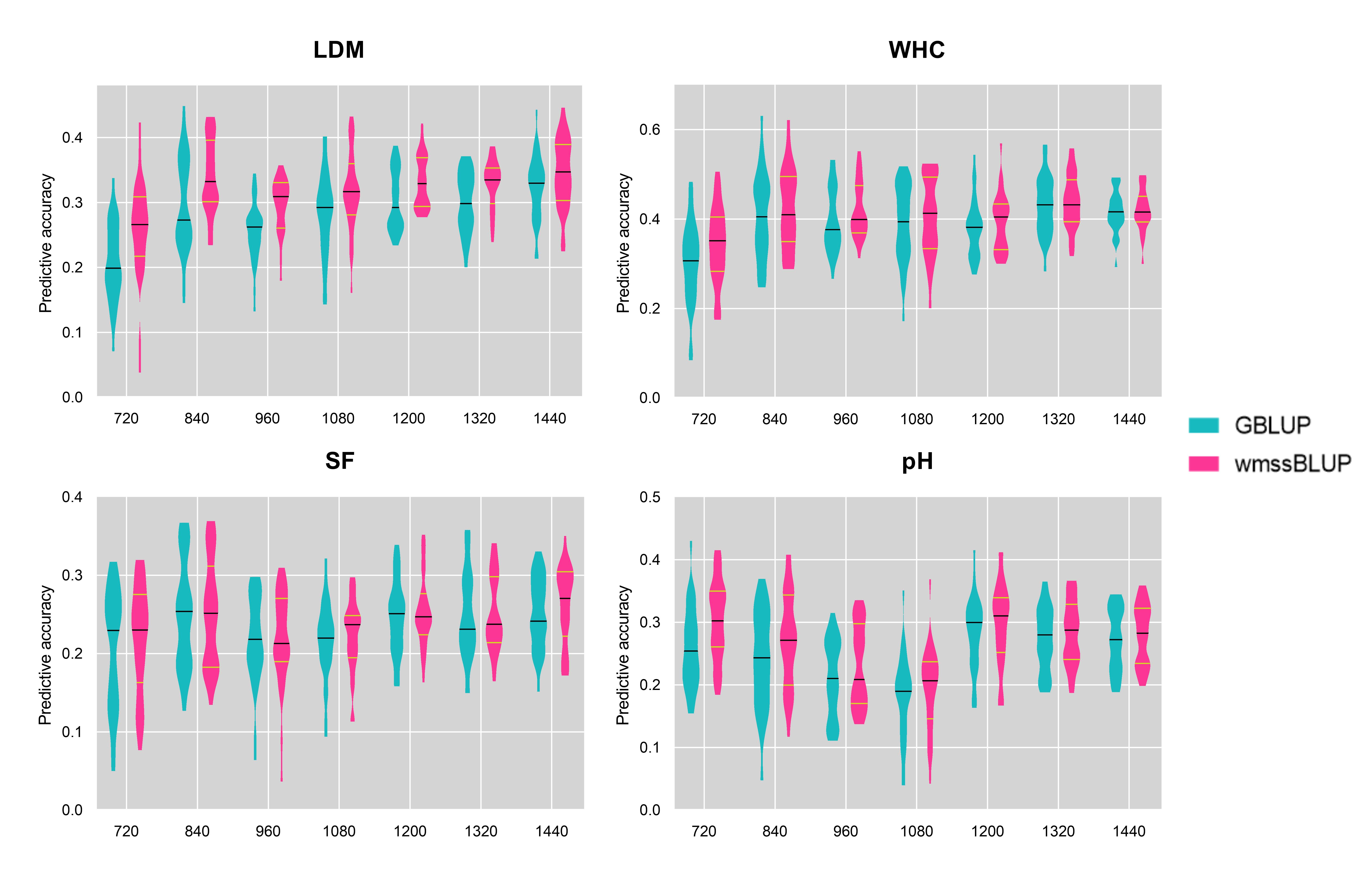

Supplement: Supplementary file 2 — Additional file 2: Fig. S2. The comparison of the accuracy of wmssBLUP and GBLUP in different population scales. [file 40104_2022_756_MOESM2_ESM.tif]
